# Prevalence, clinical significance, and persistence of autoantibodies in COVID-19

**DOI:** 10.1186/s12985-023-02191-z

**Published:** 2023-10-16

**Authors:** Se Ju Lee, Taejun Yoon, Jang Woo Ha, Jinnam Kim, Ki Hyun Lee, Jung Ah Lee, Chang Hyup Kim, Sang-Won Lee, Jung Ho Kim, Jin Young Ahn, Nam Su Ku, Jun Yong Choi, Joon-Sup Yeom, Su Jin Jeong

**Affiliations:** 1 Division of Infectious Diseases, Department of Internal Medicine and AIDS Research Institute, Yonsei University College of Medicine, Seoul, Republic of Korea; 2 Division of Infectious Diseases, Department of Internal Medicine, Inha University College of Medicine, Incheon, Republic of Korea; 3 Department of Medical Science, BK21 Plus Project, Yonsei University College of Medicine, Seoul, Republic of Korea; 4 Division of Rheumatology, Department of Internal Medicine, Yonsei University College of Medicine, Seoul, Republic of Korea

**Keywords:** Anti-nuclear antibody, Anti-phospholipid antibody, Autoantibody, COVID-19

## Abstract

**Background:**

Interest in complications and sequelae following Coronavirus disease 2019 (COVID-19) is increasing. Several articles have reported COVID-19-associated autoimmune diseases and the association between autoantibodies and the severity of COVID-19. Thromboembolic complications are frequent in patients with COVID-19, and the anti-phospholipid antibodies (aPL) is frequently detected. We conducted this study to investigate the prevalence, clinical significance, and persistence of anti-nuclear antibodies (ANA) and aPLs in COVID-19.

**Methods:**

We enrolled patients diagnosed with COVID-19 with oxygen demand and admitted to a tertiary hospital in South Korea between July 2020 and March 2022. ANA and aPLs levels were assessed using an immunoassay kit.

**Results:**

A total of 248 patients were enrolled in the study. Among them, five patients were ANA-positive, and 41 were aPL-positive (IgM anti-cardiolipin (aCL) antibody in seven patients, IgG aCL in seven patients, IgM anti-β2Glycoprotein1 antibody (aβ2-GPI) in 32 patients, and IgG aβ2-GPI in one patient). Two of five ANA-positive patients, 13 of 32 IgM aβ2-GPI-positive patients, 5 of 7 IgM aCL-positive patients, and 2 of 7 IgG aCL-positive patients were eligible for follow-up analysis, and 100%, 69.2%, 40%, and 50% of the patients remained autoantibody-positive, respectively. There were no differences in clinical outcomes between the autoantibody-positive and autoantibody-negative groups, except for the IgG aCL group showing a tendency for worse outcomes.

**Conclusion:**

A significant proportion of COVID-19 patients with oxygen demand were autoantibody-positive, and autoantibodies persisted for several months after symptom onset. Whether these autoantibodies are related to long-term sequelae in COVID-19 patients requires further investigation.

## Introduction

Severe acute respiratory syndrome coronavirus-2 has affected numerous patients worldwide; accordingly, interest in complications and sequelae following Coronavirus disease 2019 (COVID-19) is increasing [1]. A significant portion of COVID-19 patients experiences long COVID, defined as a new, returning, or ongoing health problem after COVID-19 [2]. Several mechanisms have been suggested to cause long COVID, one of which is autoimmunity [3].

Previously, there have been several studies demonstrating the association of autoimmune disorders with viral infection [4, 5]. Molecular mimicry, bystander activation, and epitope spreading have been proposed as mechanisms of autoimmunity after infection. Since a hyperinflammatory status characterizes COVID-19, that is, cytokine release syndrome, autoimmunity after COVID-19 is assumed to be induced through a similar mechanism [6]. Several articles have reported COVID-19-associated autoimmune diseases such as immune thrombocytopenia, systemic lupus erythematosus (SLE), and systemic rheumatoid disease [6, 7]. In addition, several studies have suggested the detection of autoantibodies after COVID-19 and have demonstrated the association between autoantibodies and the severity of COVID-19 [8, 9].

Thromboembolic complications are frequent in patients with COVID-19, and several studies have found that anti-phospholipid antibodies (aPL) are frequently detected in COVID-19 [10–14]. However, the clinical significance and persistence of these autoantibodies have not yet been clearly established. Therefore, we conducted this study to investigate the rate, clinical significance, and persistence of antinuclear antibodies (ANA) and aPLs in COVID-19.

## Methods

We enrolled patients diagnosed with COVID-19 who were admitted to Severance Hospital between July 2020 and March 2022. This hospital has been running a critical care unit for critically ill COVID-19 patients in South Korea during the pandemic. This study was approved by the Institutional Review Board of Yonsei University Health System Clinical Trial Centre (4-2020-0076). Written informed consent was obtained from all patients at the time of blood sampling.

Patients were included according to the following criteria: (1) older than 17 years, (2) diagnosed with COVID-19 and admitted to Severance Hospital, and (3) blood samples collected between 14 and 30 days after symptom onset.

Patients without oxygen demands or with autoimmune disease were excluded, and COVID-19 was diagnosed using real-time reverse transcriptase polymerase chain reaction (PCR) tests.

### Sample collection

Blood samples were collected from the study population between 14 and 30 days after symptom onset and on the day of outpatient follow-up. Sera were isolated from whole blood and stored at -70°C on the day of blood sampling.

Among patients confirmed to be autoantibody-positive, the persistence of autoantibodies was measured using follow-up blood samples during outpatient follow-up after discharge.

### ANA and aPL measurement

ANAs were assessed in stored serum samples using the ANA Screen 11 enzyme-linked immunosorbent assay (ELISA) kit (EUROIMMUN, Lübeck, Germany). IgG and IgM anti-β2Glycoprotein1 antibodies (aβ2-GPI) and anti-cardiolipin (aCL) antibodies were measured using ELISA kits (EUROIMMUN, Lübeck, Germany). All assays were performed according to the manufacturer’s instructions, and the cutoff value for positivity was 20 RU/mL.

### Variables and definitions

All relevant clinical and laboratory data were collected from the electronic medical records. Laboratory tests were performed according to the index date of each patient. The index date was defined as the day blood samples were collected between 14 and 30 days after the onset of symptoms. The severity of COVID-19 on the index date was classified according to the 8-category National Institute of Allergy and Infectious Disease Ordinal Scale (NIAID-OS). Patients with a NIAID-OS score of six or more were classified as having severe COVID-19, and those with a score of less than six as having mild COVID-19. The Charlson comorbidity index was calculated at admission to classify the patients according to their overall comorbidities. The Sequential Organ Failure Assessment (SOFA) score was used to measure organ dysfunction severity. Thromboembolic complications were defined as pulmonary thromboembolism, venous thromboembolism, ischaemic stroke, and systemic arterial embolism confirmed by imaging after the diagnosis of COVID-19.

### Statistical analysis

Differences in patient characteristics and outcomes between the two groups were assessed using the chi-square test or Fisher’s exact test for categorical variables and the t-test or Wilcoxon rank-sum test for continuous variables. Continuous variables were checked for normal distribution using the Shapiro-Wilk test. Logistic regression analysis was performed to assess the factors associated with autoantibodies in COVID-19. Variables with P < 0.1 in univariate analyses and with clinical relevance were entered into a multivariable model. Statistical significance was set at P < 0.05. All statistical analyses were performed using R V.4.0.5 (The R Foundation for Statistical Computing, Vienna, Austria).

## Results

**Fig. 1:**
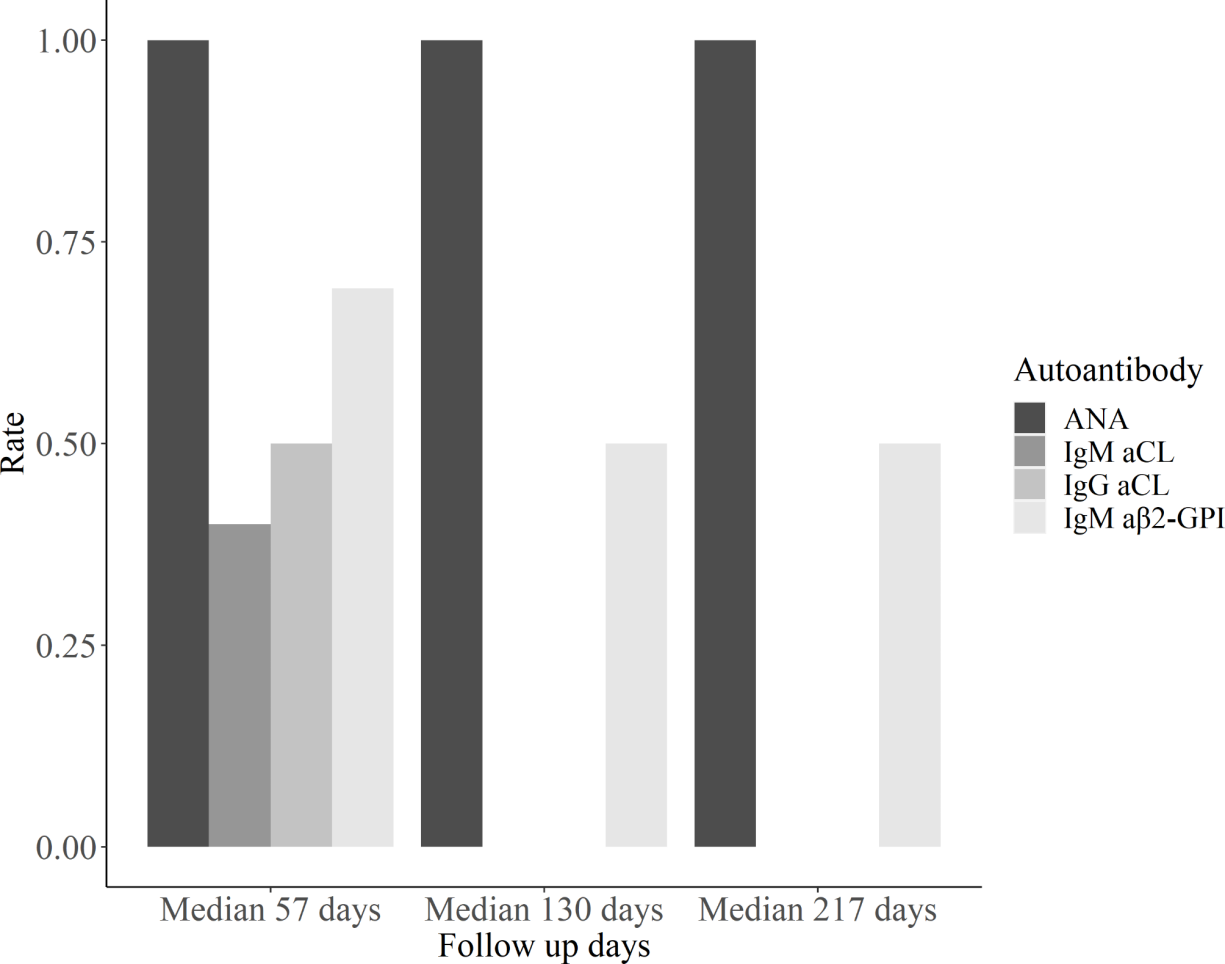
Flow chart of the study population. Abbreviations: ANA, antinuclear antibodies; aCL, anti-cardiolipin; aβ2-GPI, β2Glycoprotein1

A total of 248 patients were enrolled in the study. Among these, ANA and aPLs were found in 46 (18.5%) patients (Fig. 1). Five patients were ANA-positive, and 41 were aPL-positive (IgM aCL for seven patients, IgG aCL for seven patients, IgM aβ2-GPI for 32 patients, and IgG aβ2-GPI for one patient). Five patients showed both IgM aCL and IgM aβ2-GPI, and one showed IgG aCL and IgM aβ2-GPI. None of the patients tested positive for both ANA and aPL.

Table 1 compares the characteristics of the autoantibody-positive and autoantibody-negative patients. The two groups did not show significant differences in age, sex, comorbidities, or severity of COVID-19. Autoantibody-positive group showed significantly higher d-dimer (624.0 ng/mL; interquartile range (IQR), 306.0-2166.0 and 353.0 ng/mL; IQR 219.0-916.0, P = 0.005). The proportion of current and past smokers tended to be higher in the autoantibody-positive group (P = 0.058). Clinical outcomes such as the rate of in-hospital mortality, thromboembolic complications, invasive mechanical ventilation (IMV), and application of extracorporeal membrane oxygenation were not significantly different between the two groups. Multivariable logistic regression analysis showed that a higher D-dimer level (odds ratio (OR) 1.03 for every 100 ng/mL, 95% confidence interval (CI) 1.01–1.05, P = 0.002) was associated with autoantibodies in COVID-19 (Table 2).

**Table 1:** Comparing characteristics between autoantibody-positive and -negative patients

	Autoantibody-positive (n = 46)	Autoantibody-negative (n = 202)	*P* Value
Age, y	67.0 (56.0–76.0)	68.0 (57.0–75.0)	0.964
Sex, male, No.	33 (71.7)	129 (63.9)	0.400
Smoking status, No.			**0.058**
Current	6 (13.0)	13 (6.4)	
Previous	13 (28.3)	36 (17.8)	
Never	27 (58.7)	153 (75.7)	
Comorbidities, No.			
Hypertension	20 (43.5)	105 (52.0)	0.380
Diabetes mellitus	18 (39.1)	74 (36.6)	0.883
Coronary artery disease	3 (6.5)	30 (14.9)	0.207
Heart failure	1 (2.2)	11 (5.4)	0.581
Peripheral artery disease	0	5 (2.5)	0.619
COPD	3 (6.5)	18 (8.9)	0.817
Chronic kidney disease	3 (6.5)	31 (15.3)	0.183
Cerebrovascular accident	1 (2.2)	21 (10.4)	0.138
Solid cancer	6 (13.0)	35 (17.3)	0.627
Chronic liver disease	2 (4.3)	17 (8.4)	0.529
Connective tissue disease	0	0	> 0.99
Charlson comorbidity index	3.5 (2.0–5.0)	3.0 (2.0–5.0)	0.801
COVID-19 Severity at sample date			0.354
Mild	28 (60.9)	105 (52.0)	
Severe	18 (39.1)	97 (48.0)	
Laboratory data			
White blood cell, 10^3^/µL	8.3 (6.5–11.0)	8.1 (6.0-11.4)	0.505
Lymphocyte, 10^3^/µL	1.1 (0.7–1.6)	0.9 (0.4–1.5)	0.126
Monocyte, 10^3^/µL	0.5 (0.3–0.7)	0.4 (0.3–0.6)	0.160
Hemoglobin, g/dL	11.6 (9.4–13.1)	10.6 (9.0-12.5)	0.157
Platelet count, 10^3^/µL	233.0 (167.0-309.0)	205.0 (131.0-287.0)	0.157
International normalized ratio	1.0 (0.9–1.2)	1.0 (0.9–1.1)	0.313
aPTT, sec	28.5 (25.9–33.8)	28.4 (26.3–31.9)	0.685
Fibrinogen, mg/dL	330.0 (220.0-404.0)	280.0 (228.0-376.0)	0.441
D-dimer, ng/mL	624.0 (306.0-2166.0)	353.0 (219.0-916.0)	**0.005**
Aspartate aminotransferase, IU/L	32.0 (20.0–46.0)	30.0 (21.0–47.0)	0.904
Alanine aminotransferase, IU/L	37.0 (25.0–68.0)	33.0 (21.0–63.0)	0.505
Total bilirubin, mg/dL	0.6 (0.5–0.8)	0.6 (0.5–0.8)	0.615
Serum albumin, mg/dL	3.2 ± 0.5	3.2 ± 0.5	0.961
Blood urea nitrogen, mg/dL	19.8 (14.8–29.5)	19.2 (14.8–28.0)	0.874
Creatinine, mg/dL	0.7 (0.6–0.8)	0.7 (0.5–0.9)	0.693
Lactate dehydrogenase, IU/L	304.5 (245.0-451.0)	327.0 (257.0-398.0)	0.770
Ferritin, ng/mL	391.9 (160.2-704.7)	431.8 (201.6-798.5)	0.446
C-reactive protein, mg/L	4.6 (1.6–13.9)	4.2 (1.1–25.2)	0.525
Procalcitonin, ng/mL	0.1 (0.0-0.2)	0.0 (0.0-0.2)	0.295
Arterial lactate, mmol/L	1.5 (1.2-2.0)	1.5 (1.1–1.9)	0.323
Plasma interleukin 6, pg/mL	24.0 (13.1–168.0)	73.0 (20.2–248.0)	0.131
Outcomes			
In-hospital mortality, No.	10 (21.7)	51 (25.2)	0.757
Thromboembolism, No.	4 (8.7)	13 (6.4)	0.823
Mechanical ventilation	21 (45.7)	82 (40.6)	0.644
ECMO support, No.	1 (2.2)	6 (3.0)	> 0.99
CRRT, No.	3 (6.5)	12 (5.9)	> 0.99
Length of stay, d	19.0 (13.0–27.0)	18.0 (13.0–30.0)	0.619

Abbreviations: IQR, Interquartile range; COPD, Chronic Obstructive Pulmonary Disease; ECMO, Extracorporeal membrane oxygenation; CRRT, Continuous renal replacement therapy

Data are expressed as the mean ± standard deviation, median (interquartile range), or number (%)

**Table 2:** Multivariable analysis for risk factors of antiantibodies in COVID-19

	OR (95% CI)	*P* Value
D-dimer (for every 100 ng/mL)	1.03 (1.01–1.05)	**0.002**
Smoking status		
Current	Reference	
Previous	0.81 (0.25–2.75)	0.721
Never	0.37 (0.13–1.14)	0.068

Abbreviations: OR, Odds ratio; CI, Confidence interval

A comparison of clinical characteristics between autoantibody-negative patients and patients with each autoantibody is shown in Table 3. The IgM aCL/IgM aβ2-GPI-positive group showed a significantly lower SOFA score and a higher PaO2/FiO2 (P/F) ratio than the autoantibody-negative group. The duration of steroid administration and proportion of second immunomodulator administration were significantly lower in the IgM aβ2-GPI group than in the autoantibody-negative group. When comparing clinical outcomes between autoantibody-negative patients and patients with each autoantibody, there were no significant differences(Table 4). Nevertheless, the rates of in-hospital mortality (66.7% and 25.2%, P = 0.072), thromboembolic events (33.3% and 6.4%, P = 0.087), and the need for continuous renal replacement therapy (CRRT) during treatment (33.3% and 5.9%, P = 0.07) showed a higher trend in IgG aCL-positive patients.

**Table 3:** Comparison of clinical characteristics between autoantibody-negative patients and ANA, IgM aβ2-GPI, IgG aCL, and IgM aCL/IgM aβ2-GPI patients

	Control (n = 202)	ANA (n = 5)	IgM aβ2-GPI (n = 26)	IgG aCL (n = 6)	IgM aCL/IgM aβ2-GPI (n = 5)	*P*	*P`*	*P``*	*P```*
Age, y	68.0 (57.0–75.0)	71.0 (60.0–83.0)	65.5 (56.0–80.0)	71.5 (68.0–76.0)	54.0 (50.0–64.0	0.452	0.869	0.231	0.101
Sex, male, No.	129 (63.9)	4 (80.0)	17 (65.4)	6 (100.0)	4 (80.0)	0.786	> 0.99	0.163	0.786
Comorbidities, No.									
Hypertension	105 (52.0)	2 (40.0)	9 (34.6)	4 (66.7)	2 (40.0)	0.939	0.145	0.768	0.939
Diabetes mellitus	74 (36.6)	2 (40.0)	9 (34.6)	3 (50.0)	1 (20.0)	> 0.99	> 0.99	> 0.811	0.769
Coronary artery disease	30 (14.9)	1 (20.0)	2 (7.7)	0	0	> 0.99	0.491	0.667	0.773
Heart failure	11 (5.4)	0	1 (3.8)	0	0	> 0.99	> 0.99	> 0.99	> 0.99
Peripheral artery disease	5 (2.5)	0	0	0	0	> 0.99	0.920	> 0.99	> 0.99
COPD	18 (8.9)	1 (20.0)	2 (7.7)	0	0	0.949	0.669	0.977	> 0.99
Chronic kidney disease	31 (15.3)	0	2 (7.7)	1 (16.7)	0	0.752	0.454	> 0.99	0.752
Cerebrovascular accident	21 (10.4)	0	1 (3.8)	0	0	0.991	> 0.99	0.884	0.991
Solid cancer	35 (17.3)	2 (40.0)	1 (3.8)	1 (16.7)	0	0.474	0.137	> 0.99	0.677
Chronic liver disease	17 (8.4)	1 (20.0)	1 (3.8)	0	0	0.917	0.669	> 0.99	> 0.99
Charlson comorbidity index	3.0 (2.0–5.0)	4.0 (2.0–6.0)	3.0 (2.0–5.0)	4.0 (2.0–5.0)	1.0 (1.0–2.0)	0.648	0.548	0.625	**0.05**
COVID-19 Severity at sample date						0.432	0.734	> 0.99	> 0.99
Mild	105 (52.0)	4 (80.0)	15 (57.7)	3 (50.0)	3 (60.0)				
Severe	97 (48.0)	1 (20.0)	11 (42.3)	3 (50.0)	2 (40.0)				
COVID-19 related treatment, No.									
Steroid	198 (98.0)	5 (100.0)	24 (92.3)	6 (100.0)	5 (100.0)	> 0.99	0.288	> 0.99	> 0.99
High dose steroid ^a^	137 (67.8)	4 (80.0)	15 (57.7)	4 (66.7)	2 (40.0)	0.927	0.418	> 0.99	0.409
Steroid cumulative dose, mg	115.0 (54.0-169.0)	96.0 (87.5-136.9)	99.4 (18.0-134.0)	93.6 (46.0-142.5)	94.0 (34.5-110.5)	0.8	0.095	0.5	0.43
Steroid duration, d	16.0 (9.0–21.0)	8.0 (7.0–18.0)	12.5 (6.0–17.0)	15.5 (8.0–22.0)	15.0 (10.0–20.0)	0.203	**0.027**	0.855	0.812
Remdesivir	170 (84.2)	5 (100.0)	21 (80.8)	4 (66.7)	4 (80.0)	0.732	0.874	0.561	> 0.99
Iimmunomodulatory agents						0.279	**0.009**	**0.035**	> 0.99
Baricitinib	18 (8.9)	0	0	0	1 (20.0)				
Tocillizumab	120 (59.4)	5 (100.0)	10 (38.5)	1 (16.7)	2 (40.0)				
SOFA score	2.0 (1.0–5.0)	2.0 (2.0–3.0)	2.0 (0.0–3.0)	1.5 (0–6.0)	1.0 (1.0–1.0)	0.893	0.285	0.717	**0.041**
PaO2/FiO2 ratio	266.4 (176.4-383.6)	264.9 (247.3-302.4)	357.1 (205.5-404.8)	374.0 (263.5–432)	389.0 (361.5-395.7)	0.642	0.171	0.256	**0.037**

Abbreviations: ANA, antinuclear antibodies; aβ2-GPI, anti-β2 Glycoprotein1 antibody; aCL, anti-Cardiolipin antibody; *P*, ANA-positive and control group; *P`*, IgM aβ2-GPI-positive and control group; *P``*, IgG aCL-positive and control group; *P```*, IgM aCL/IgM aβ2-GPI-positive and control group; IQR, interquartile range; COPD, Chronic Obstructive Pulmonary Disease; SOFA, Sequential Organ Failure Assessment

Data are expressed as median (interquartile range) or number (%)

^a^ higher than dexamethasone 6mg

**Table 4:** Comparison of clinical outcomes between autoantibody-negative patients and ANA, IgM aβ2-GPI, IgG aCL, and IgM aCL/IgM aβ2-GPI patients

	Control group (n = 202)	ANA (n = 5)	IgM aβ2-GPI (n = 26)	IgG aCL (n = 6)	IgM aCL/IgM aβ2-GPI (n = 5)	*P*	*P`*	*P``*	*P```*
In-hospital mortality, No.	51 (25.2)	1 (20.0)	5 (19.2)	4 (66.7)	0	> 0.99	0.668	**0.072**	0.442
Thromboembolism, No.	13 (6.4)	0	2 (7.7)	2 (33.3)	0	> 0.99	> 0.99	**0.087**	> 0.99
Mechanical ventilation, No.	82 (40.6)	0	11 (42.3)	5 (83.3)	3 (60.0)	0.171	> 0.99	0.095	0.681
ECMO support, No.	6 (3.0)	0	1 (3.8)	0	0	> 0.99	> 0.99	> 0.99	> 0.99
CRRT, No.	12 (5.9)	0	1 (3.8)	2 (33.3)	0	> 0.99	> 0.99	**0.07**	> 0.99
Length of stay, median, d	18.0 (13.0–30.0)	13.0 (12.0–17.0)	18.0 (13.0–25.0)	2.0 (22.0–28.0)	14.0 (13.0–21.0)	0.13	0.694	0.426	0.412

Abbreviations: ANA, antinuclear antibodies; aβ2-GPI, anti-β2 Glycoprotein1 antibody; aCL, anti-Cardiolipin antibody; CRRT, Continuous renal replacement therapy; *P*, ANA-positive and control group; *P`*, IgM aβ2-GPI-positive and control group; *P``*, IgG aCL-positive and control group; *P```*, IgM aCL/IgM aβ2-GPI-positive and control group; IQR, interquartile range; ECMO, Extracorporeal membrane oxygenation

Data are expressed as median (interquartile range) or number (%)

**Fig. 2:**
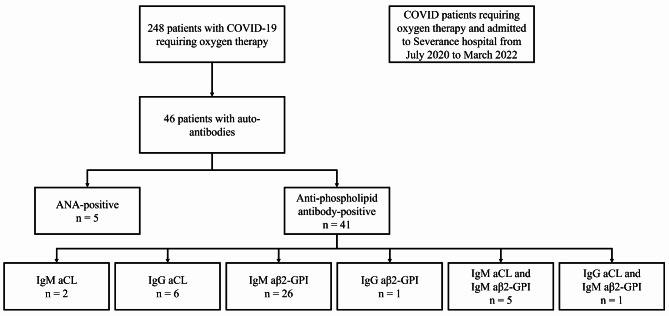
Follow up analysis for autoantibody-positive patients. Abbreviations: ANA, antinuclear antibodies; aCL, anti-cardiolipin; aβ2-GPI, β2Glycoprotein1

Among the 46 autoantibody-positive patients, 22 were eligible for at least one follow-up measurement (Fig. 2). Two ANA-positive, 13 IgM aβ2-GPI-positive, 5 IgM aCL-positive, and 2 IgG aCL-positive patients were measured for autoantibodies 53 days after symptom onset, and 100%, 69.2%, 40%, and 50% of the patients remained autoantibody-positive, respectively. At a median of 130 days after symptom onset, one ANA-positive, four IgM aβ2-GPI-positive, and two IgM aCL-positive patients were eligible for follow-up analysis, and 100%, 50%, and 0% of the patients maintained autoantibodies, respectively. One ANA-positive patient and one of the two IgM aβ2-GPI positive patients were consistently autoantibody-positive at a median follow-up of 217 days.

## Discussion

As COVID-19 continues to spread, the expression of autoimmunity and clinical significance of autoantibodies after COVID-19 remain a global interest and concern. A dysregulated host response characterizes the immune response in COVID-19, also known as the cytokine release syndrome [15]. The hyperinflammatory response or persistent infection of COVID-19 might induce autoimmunity, consistent with previous studies suggesting that the presence of autoantibodies in COVID-19 is associated with disease severity and worse prognosis [8, 16]. In this study, 18.5% of the COVID-19 patients with oxygen demand were ANA- or aPL-positive between 14 and 30 days after symptom onset. However, the presence of autoantibodies was not associated with a worse prognosis of COVID-19. Uniquely, the IgG aCL-positive group, among the autoantibody-positive patients, tended to show a higher severity than the autoantibody-negative group. Since the difference in the clinical implications of aPLs is unclear, the higher severity of the IgG aCL group is a significant result of this study, and further studies are needed [17].

Whether aPL that appears during COVID-19 infection is a risk factor for thrombosis has not been clearly established. Zuo et al. demonstrated that aPL induced thrombosis in animal models [11]. In contrast, a study by Borghi et al. revealed that antibodies from COVID-19 patients had different epitope specificity from aPL [18]. Several other studies also did not reveal an association between thrombosis and aPL [19]. In this study, the rate of thromboembolic events did not differ between the aPL-positive and aPL-negative groups. It was also assumed that multiple aPLs might increase the risk of thrombosis [19]. However, in our study, there were no thromboembolic events or in-hospital mortality among the six double aPL-positive patients. Only the IgG aCL-positive group had more thromboembolic events. Thromboembolic events might be more prevalent in the IgG aCL-positive group, as this group showed higher severity than the others. However, whether IgG aCL from COVID-19 has distinct features from other aPLs requires further research.

Several studies have been conducted on autoantibodies after COVID-19, but few have investigated the persistence of autoantibodies [12, 19, 20]. A previous study by Devreese et al. demonstrated transient aPLs in COVID-19 with a predominantly negative result of aPL by repeated test after one month [20]. Conversely, Vollmer et al. reported the presence of ANA and aPLs at a significant rate after 3–6 months [12]. In our study, a significant proportion of the autoantibody-positive patients between 14 and 30 days after symptom onset showed persistence of autoantibodies and one patient even on the 235th day. Although thrombosis or pregnancy morbidity is required to diagnose anti-phospholipid syndrome (APS), it is noteworthy that aPL persisted for more than 12 weeks in many cases in our study [21]. In these patients, monitoring of thromboembolic events through long-term follow-up should be considered.

In the logistic regression analysis to identify the factors associated with autoantibodies in this study, high d-dimer levels were associated with autoantibodies. A higher d-dimer level is associated with a poor prognosis for COVID-19 [22]. Accordingly, the association of higher d-dimer levels with the presence of autoantibodies might imply that the hyperinflammatory response, a characteristic of COVID-19, is associated with the development of aPLs, as proposed by previous studies. For patients with high d-dimer levels, measuring the presence of aPLs or careful monitoring for thromboembolic complications should be considered. Although statistical significance was not achieved, never-smokers showed a protective effect against the presence of autoantibodies in the logistic regression analysis. Smoking is known to be associated with autoimmune diseases such as SLE and APS; therefore, a study with a larger population might demonstrate the association between smoking and autoantibodies in COVID-19 [23, 24].

This study had several limitations. First, because this study measured autoantibodies using blood samples between 14 and 30 days after symptom onset, patients who died early due to the rapid progression of COVID-19 could not be included. However, since the purpose of this study was to measure autoantibodies in terms of the long-term complications of COVID-19, we considered the measurement of autoantibodies using blood samples between 14 and 30 days after symptom onset to meet the goal of this study. Second, long-term autoantibody measurement is a strength of our study, but not many patients were eligible for long-term measurement, as this was a single-institution study. Further studies with more patients are required to clarify the persistence of autoantibodies after COVID-19. Third, an imaging study was not routinely performed in all patients to confirm thromboembolic complications, but only when clinically suspected; thus, thromboembolic complications could have been underestimated. However, our institution routinely has applied prophylactic anticoagulation to COVID-19 patients with oxygen demand unless contraindicated and carefully monitored in a critical care unit setting; accordingly, missed thromboembolic cases would not cause significant deviation in the results of this study.

In conclusion, our study demonstrated that a significant proportion of COVID-19 patients with oxygen demand showed the presence of autoantibodies, many of which were maintained several months after symptom onset, and the association of autoantibodies with higher d-dimer levels. Whether these autoantibodies are related to long-term sequelae in COVID-19 patients requires further investigation.

## Data Availability

The data used and/or analyzed during the current study are available from the corresponding author on reasonable request.
